# Supplementary material for: Resolving the full spectrum of human genome variation using Linked-Reads
Source: Genome Res. 2019 Apr;29(4):635–45. doi: 10.1101/gr.234443.118 (PMC6442396; doi:10.1101/gr.234443.118)
Supplement: Supplemental Material [file supp_gr.234443.118_Supplemental_longranger-2.2.zip › longranger-2.2.tar.bz2!/lr2.2-public/lib/stan/cmdstan/stan/doc/api/html/math_2matrix_2trace__inv__quad__form__ldlt_8hpp_source.html]

Stan: src/stan/math/matrix/trace\_inv\_quad\_form\_ldlt.hpp Source File


|  |  |
| --- | --- |
|  | Stan  2.5.0  probability, sampling & optimization |


- Main Page
- Related Pages
- Namespaces
- Classes
- Files

- File List
- File Members

All Classes Namespaces Files Functions Variables Typedefs Enumerations Enumerator Friends Macros Pages

- src
- stan
- math
- matrix

trace\_inv\_quad\_form\_ldlt.hpp

Go to the documentation of this file.

1 #ifndef STAN\_\_MATH\_\_MATRIX\_\_TRACE\_INV\_QUAD\_FORM\_LDLT\_HPP

2 #define STAN\_\_MATH\_\_MATRIX\_\_TRACE\_INV\_QUAD\_FORM\_LDLT\_HPP

3

4 #include <stan/math/matrix/Eigen.hpp>

5 #include <stan/math/matrix/LDLT\_factor.hpp>

6 #include <stan/math/error\_handling/matrix/check\_multiplicable.hpp>

7 #include <stan/math/matrix/mdivide\_left\_ldlt.hpp>

8 #include <stan/math/matrix/trace.hpp>

9 #include <stan/math/matrix/transpose.hpp>

10 #include <stan/math/matrix/multiply.hpp>

11

12 namespace stan {

13  namespace math {

14

15  /\*

16  \* Compute the trace of an inverse quadratic form. I.E., this computes

17  \* trace(B^T A^-1 B)

18  \* where the LDLT\_factor of A is provided.

19  \*/

20  template <typename T1, typename T2, int R2,int C2,int R3,int C3>

21  inline typename

22  boost::enable\_if\_c<!stan::is\_var<T1>::value &&

23  !stan::is\_var<T2>::value,

24  typename boost::math::tools::promote\_args<T1,T2>::type>::type

25  trace\_inv\_quad\_form\_ldlt(const stan::math::LDLT\_factor<T1,R2,C2> &A,

26  const Eigen::Matrix<T2,R3,C3> &B) {

27  stan::math::check\_multiplicable("trace\_inv\_quad\_form\_ldlt(%1%)",A,"A",

28  B,"B",(double\*)0);

29

30  return trace(multiply(transpose(B),mdivide\_left\_ldlt(A,B)));

31  }

32  }

33 }

34

35 #endif

mdivide\_left\_ldlt.hpp

stan::math::trace\_inv\_quad\_form\_ldlt

boost::enable\_if\_c<!stan::is\_var< T1 >::value &&!stan::is\_var< T2 >::value, typename boost::math::tools::promote\_args< T1, T2 >::type >::type trace\_inv\_quad\_form\_ldlt(const stan::math::LDLT\_factor< T1, R2, C2 > &A, const Eigen::Matrix< T2, R3, C3 > &B)

**Definition:** trace\_inv\_quad\_form\_ldlt.hpp:25

check\_multiplicable.hpp

stan::is\_var

**Definition:** traits.hpp:421

stan::math::mdivide\_left\_ldlt

Eigen::Matrix< typename boost::math::tools::promote\_args< T1, T2 >::type, R1, C2 > mdivide\_left\_ldlt(const stan::math::LDLT\_factor< T1, R1, C1 > &A, const Eigen::Matrix< T2, R2, C2 > &b)

Returns the solution of the system Ax=b given an LDLT\_factor of A.

**Definition:** mdivide\_left\_ldlt.hpp:24

transpose.hpp

trace.hpp

stan::math::check\_multiplicable

bool check\_multiplicable(const char \*function, const T1 &y1, const char \*name1, const T2 &y2, const char \*name2, T\_result \*result)

**Definition:** check\_multiplicable.hpp:15

stan::math::transpose

Eigen::Matrix< T, C, R > transpose(const Eigen::Matrix< T, R, C > &m)

**Definition:** transpose.hpp:12

stan::math::LDLT\_factor

**Definition:** LDLT\_factor.hpp:16

Eigen.hpp

stan::math::trace

T trace(const Eigen::Matrix< T, Eigen::Dynamic, Eigen::Dynamic > &m)

Returns the trace of the specified matrix.

**Definition:** trace.hpp:20

stan::math::multiply

boost::enable\_if\_c< boost::is\_arithmetic< T >::value, Eigen::Matrix< double, R, C > >::type multiply(const Eigen::Matrix< double, R, C > &m, T c)

Return specified matrix multiplied by specified scalar.

**Definition:** multiply.hpp:25

multiply.hpp

LDLT\_factor.hpp


---

[ Stan Home Page ]

*© 2011–2014,
Stan Development Team.*
